# Patients with Borderline Personality Disorder in Emergency Departments

**DOI:** 10.3389/fpsyt.2017.00136

**Published:** 2017-08-04

**Authors:** Untara Shaikh, Iqra Qamar, Farhana Jafry, Mudasar Hassan, Shanila Shagufta, Yassar Islamail Odhejo, Saeed Ahmed

**Affiliations:** ^1^Liaquat University of Medical & Health Sciences, Jamshoro, Pakistan; ^2^Nassau University Medical Center, East Meadow, NY, United States; ^3^Punjab Medical College, Faisalabad, Pakistan; ^4^NYU Langone Medical Center, New York, NY, United States; ^5^A & L Physicians, New York, NY, United States; ^6^Kings County Hospital Center, Brooklyn, NY, United States

**Keywords:** borderline personality disorder, emergency psychiatry, psychotherapy, cluster B personality disorders, psychosocial issues, impulsivity, aggression, suicidality

## Abstract

Borderline personality disorder (BPD) patients, when in crisis, are frequent visitors of emergency departments (EDs). When these patients exhibit symptoms such as aggressiveness, impulsivity, intense anxiety, severe depression, self-harm, and suicidal attempts or gestures, diagnosis, and treatment of the BPD becomes challenging for ED doctors. This review will, therefore, outline advice to physicians and health-care providers who face this challenging patient population in the EDs. Crisis intervention should be the first objective of clinicians when dealing with BPD in the emergency. For the patients with agitation, symptom-specific pharmacotherapy is usually recommended, while for non-agitated patients, short but intensive psychotherapy especially dialectical behavior therapy (DBT) has a positive effect. Although various psychotherapies, either alone or integrated, are preferred modes of treatment for this group of patients, the effects of psychotherapies on BPD outcomes are small to medium. Proper risk management along with developing a positive attitude and empathy toward these patients will help them in normalizing in an emergency setting after which treatment course can be decided.

## Methodology

Using the Preferred Reporting Items for Systematic Reviews and Meta-Analyses methodology (1), a search for relevant published literature was done using PubMed. The key words and phrases used together with Boolean operators included: “borderline personality disorder in emergency department” (Mesh), “borderline personality disorder pharmacotherapy and psychotherapy” (Mesh), “dialectical behavior therapy, cognitive behavioral therapy in borderline personality disorder” (Mesh), borderline personality disorder and cluster B personality disorders (Mesh), “borderline personality disorder and impulsivity, aggression, suicidality” (Mesh). Other relevant studies were found by a review of the primary studies obtained in the search as well as reference tracing of selected articles.

The inclusion and exclusion criteria were:

- Any articles that reported the patient of borderline personality disorder (BPD), crisis intervention in the Emergency departments (EDs) and beyond in terms of acute and long-term treatment plan.
- Eligible studies were included if they were observational or interventional in which pharmacotherapy or psychotherapy were investigated as immediate or follow-up treatment.
- We included both observational and interventional studies whether with control groups or not. No restrictions were placed on the control group; we included placebo, treatment as usual, or any unspecific treatment for BPD.
- Only peer-reviewed research studies, which were published in the English. Specific case studies, case letters, and gray literature as well as studies not published in English were excluded.

The above-outlined search strategy allowed for the retrieval a total of 396 articles following the removal of duplicates from various sources. The identified results were then reviewed by two independent researchers. From the 396 articles obtained, only 71 studies were relevant to the topic of review. Article relevance was found after looking at the title of the article and reading their abstracts. After a full-text review, 56 of the 71 relevant articles were found and used to extract qualitative data and summarize the findings from this literature review (Tables 1 and 2).

**Table 1:** Studies that investigated immediate crisis intervention in BPD patients.

Study	Study design	Number of patients	Treatment strategy	Results/treatment response
Philipsen et al. (69)	An open label study	14 females with acute states of strong aversive inner tension and urge to commit self-injurious behavior	75 and 150 μg of oral clonidine	After administration of clonidine in both doses, aversive inner tension, dissociative symptoms, urge to commit self-injurious behavior, and suicidal ideations significantly decreased. The peak effect was after 30–60 min

Damsa et al. (70)	Observational study	25 patients with acute agitation	Olanzapine 10 mg IM single injection	Significant improvement of agitation with good tolerance noticed 2 h after the first injection. 60% of patients required a second injection

Linehan et al. (71)	A double-blind, placebo-controlled pilot study	24 female patients with BPD	Patients received DBT for 6 months, then olanzapine or placebo	Olanzapine may promote more rapid reduction of irritability and aggression than placebo for highly irritable women with PBD

Berrino et al. (72)	A prospective cohort study	200 BPD patients; 100 received crisis intervention and 100 received treatment as usual	Crisis intervention vs. treatment as usual 1–10 days and followed up for 3 months	The results suggested that short-term intensive care at the general hospital may contribute to BPD emergency although this treatment is not considered as an alternative to structured psychiatric acute treatment

Bertsch et al. (73)	A randomized placebo-controlled double-blind group design	40 patients and 41 controls	26 IU of oxytocin or placebo as single dose	Oxytocin may decrease social threat hypersensitivity and thus reduce anger and aggressive behavior in PBD with enhanced threat-driven reactive aggression

Carvalho Fernando et al. (74)	A crossover placebo-controlled double group design	32 females with BPD and 32 healthy females	A single administration of 10 mg hydrocortisone or placebo	Acute hydrocortisone administration enhances response inhibition of face stimuli in BPD patients and healthy controls, regardless of their emotional valence

Brune (75)	A double-blind placebo-controlled study	15 PBD patients and 15 controls	Intranasal oxytocin single dose	Oxytocin was associated with less fight behavior in both groups

**Table 2:** Studies that investigate follow-up and treatment of patients with BPD.

Study	Study design	Number of patients	Treatment strategy	Results/treatment response
Hollander et al. (76)	A preliminary double-blind, placebo-controlled trial	16	Divalproex sodium vs. placebo for 10 weeks	Divalproex sodium was more effective than placebo for global symptomatology, aggression, and depression

Zanarini and Frankenburg (77)	A double-blind, placebo-controlled study	28 females	Olanzapine vs. placebo for 6 months	Olanzapine had greater effect than placebo in all symptoms except depression

Rinne et al. (78)	A randomized, placebo-controlled clinical trial	38 BPD female patients	The SSRI fluvoxamine for 6 weeks followed by a blind half-crossover for 6 weeks and an open follow-up for another 12 weeks	Fluvoxamine significantly improved rapid mood shifts in female borderline patients, but not impulsivity and aggression

Rocca et al. (79)	An open-label study	13 patients	Risperidone at low-to-moderate doses	There was a significant reduction in aggression based on Aggression Questionnaire scores

Zanarini et al. (80)	A randomized double-blind study	45 patients	Fluoxetine, olanzapine, or olanzapine–fluoxetine combination for 8 weeks	The three groups showed significant improvement of symptoms. Olanzapine monotherapy and fluoxetine–olanzapine combination were superior to fluoxetine alone

Bogenschutz and George Nurnberg et al. (81)	A randomized double group, placebo-controlled trial	40 BPD patients	Olanzapine 2.5–20 mg/day or placebo for 12 weeks	Olanzapine was found to be significantly (*p* < 0.05) superior to placebo on the CGI-BPD at endpoint

Simpson et al. (30)	A randomized, double-blind, placebo-controlled study	20 patients with BPD	All subjects received individual and group DBT followed by 40 mg/day of fluoxetine or placebo for 12 weeks	Adding fluoxetine to an efficacious psychosocial treatment does not provide any additional benefits

Villeneuve and Lemelin (82)	An open-label study	23	Quetiapine 175–400 mg/day for 12 weeks	A low dose of quetiapine was associated with a strong positive clinical impact, including improvement of impulsivity

Bellino et al. (83)	An open-label pilot study	17	Oxcarbazepine 1,200–1,500 mg/day for 12 weeks	A statistically significant response to oxcarbazepine was observed according to CGI-S and BPRS mean score No cases of significant hyponatremia or severe adverse effects were reported

Soler et al. (84)	A double-blind, placebo-controlled study	60 patients with BPD	Dialectical behavior therapy followed by olanzapine or placebo for 12 weeks	Olanzapine was associated with a statistically significant improvement over placebo in depression, anxiety, and impulsivity/aggressive behavior

Hollander et al. (85)	A double blind, placebo-controlled trial	52 BPD patients	Divalproex or placebo for 12 weeks	Divalproex was superior to placebo in reducing impulsive aggression in patients with borderline personality disorder

Tritt et al. (86)	A randomized, double-blind, placebo-controlled study	24 females with BPD	Lamotrigine or placebo for 8 weeks	Highly significant (*p* < 0.01) changes on four STAXI scales were observed on lamotrigine group

Loew et al. (87)	A double-blind, placebo-controlled study	56 patients	Topiramate titrated from 25–200 mg/day or placebo for 10 weeks	Significant changes on the somatization, interpersonal sensitivity, anxiety, hostility, phobic anxiety, and Global Severity Index scales of the Symptom Checklist were observed in the topiramate-treated subjects after 10 weeks

Nickel et al. (88)	A double-blind, placebo-controlled study	29 female patients	Topiramate or placebo for 8 weeks	Significant improvements on four subscales of the STAXI (state-anger, trait-anger, anger-out, anger-control) were observed in the topiramate-treated subjects after 8 weeks, in comparison with the placebo group

Linehan et al. (57)	A randomized controlled trial	100 women with recent suicidal attempts or self-injuring behavior	One year of DBT or 1 year of community treatment by experts	Dialectical behavior therapy was associated with better outcomes in the intent-to-treat analysis than community treatment by experts in most target areas during the 2-year treatment and follow-up period

Giesen-Bloo et al. (89)	A multicenter, randomized, two-group design trial	88 patients	Three years of either SFT or TFP with sessions twice a week	Statistically and clinically significant improvements were found for both treatments. More patients in SFT group showed significant recovery and clinical improvement

Nickel et al. (90)	A double-blind, placebo-controlled study	42 patients	15 mg/day of aripiprazole for 8 weeks	Significant changes in scores on most scales were observed in the subjects treated with aripiprazole after 8 weeks

Bellino et al. (91)	An open-label pilot study	14	Quetiapine at the dose of 200–400 mg/day for 12 weeks	Data suggested that quetiapine is effective in BPD patients specially with impulsiveness/aggressiveness-related symptoms

Clarkin et al. (92)	A multi-wave study	90 patients	Transference-focused psychotherapy, dialectical behavior therapy, or supportive treatment	Both transference-focused psychotherapy and dialectical behavior therapy were significantly associated with improvement in suicidality

Silva et al. (93)	An open label study	59 patients	Flexible doses of fluoxetine for 12 weeks	LL carriers had a better response than S carriers in the reduction of total OAS-M scores and on the aggressiveness and irritability components of the OAS-M

Bateman and Fonagy (94)	A follow-up study after randomized, controlled trial was complete by 8 years	41 patients	Mentalization-based treatment or treatment as usual for 18 months	Mentalization-based treatment showed superior results in suicidality, service use, use of medication, and global function above 60 than treatment as usual group

Adityanjee et al. (95)	An open-label pilot trial	16	Quetiapine for 8 weeks	Significant reductions in symptoms were observed in this pilot study

Blum et al. (96)	A randomized controlled trial and 1-year follow-up	124	STEPPS plus treatment as usual or treatment as usual alone	STEPPS an adjunctive group treatment, can deliver clinically meaningful improvements in borderline personality disorder-related symptoms and behaviors, enhance global functioning, and relieve depression

Clivaz et al. (27)	A case report	A 17-year-old woman with BPD, administrated to ER with panic attacks	Topiramate (TPM) at 25 mg daily for a month	Panic attacks intensity increased and disappeared after discontinuation of TPM

Pascual et al. (97)	A double-blind, placebo-controlled study	60	Ziprasidone 84.1 mg/day vs. placebo for 2 weeks	There was no statistically significant difference between ziprasidone and placebo

Van den Eynde et al. (98)	An open-label study	41	Quetiapine 100–800 mg/day for 12 weeks	The results showed that quetiapine may be effective in the treatment of impulsivity and affective symptoms in BPD.

Bellino et al. (99)	An open-label study	21 patients with BPD resistant to sertraline therapy	Aripiprazole 100–200 mg/day for 12 weeks	Aripiprazole is an efficacious and well-tolerated add-on treatment for sertraline-resistant BPD patients

McMain et al. (100)	A single blind randomized controlled study	180 patients	Dialectical behavior therapy or general psychiatric management for 1 year	Patients benefited equally from both types of treatment

Farrell et al. (62)	A randomized controlled trial	32	A group received schema-based therapy plus as usual treatment. The other received treatment as usual only	Schema-based therapy had more significant improvements that led to recovery and improved overall functioning

Reich et al. (101)	A double-blind, placebo-controlled study	28 patients	Lamotrigine or placebo for 12 weeks	Patients in the lamotrigine group had significantly greater reductions in the total Affective Lability Scale scores. Lamotrigine is an effective treatment for affective instability and for the general impulsivity characteristic of BPD

Ziegenhorn et al. (102)	A randomized, double-blind, placebo-controlled, crossover study trial	18 patients with BPD, with or without comorbid PTSD, and with a prominent hyperarousal syndrome	Clonidine or placebo	Clonidine might be a useful adjunct to pharmacotherapy in patients with BPD who have marked hyperarousal and/or sleep problems

Shafti and Shahveisi (103)	A randomized double-blind trial	28 female patients	Olanzapine or haloperidol for 8 weeks	Both groups showed significant improvement but no inter-group difference was found

Bellino et al. (104)	A pilot study	18 patients	Open-label duloxetine, 60 mg/day, for 12 weeks	A notable change was found for: BPRS, HAM-D, SOFAS, BPDSI total score, and items “impulsivity,” “outbursts of anger,” and “affective instability” and HSCL-90 SOM

Doering et al. (105)	A randomized controlled trial	104 females with BPD	Transference-focused psychotherapy or by an experienced community psychotherapist for 1 year	Transference-focused psychotherapy is more efficacious than treatment by experienced community psychotherapists in the domains of borderline symptomatology, psychosocial functioning, and personality organization. Self-harming behavior did not change in either group

Harned et al. (106)	An open label study	51 women with suicidal or self-injuring behavior	Dialectical behavior therapy for 1 year	BPD clients with and without PTSD were equally likely to eliminate the exclusionary behaviors during 1 year of DBT

Bellino et al. (104)	A randomized double group design	55 patients with BPD	Two groups: fluoxetine 20–40 mg/day plus clinical management, or fluoxetine 20–40 mg/day plus interpersonal psychotherapy adapted to BPD	Combined therapy with adapted IPT was superior to fluoxetine alone in BPD patients, concerning a few core symptoms of the disorder, anxiety, and quality of life

Zanarini et al. (107)	A randomized, double-blind, placebo-controlled study	451	Olanzapine 2.5 mg/day, olanzapine 5–10 mg/day, or placebo	Olanzapine 5–10 mg/day showed a clinically modest advantage over placebo in the treatment of overall borderline psychopathology

Zanarini et al. (108)	An open label study	472	Patients received open-label olanzapine for 12 weeks after 12 weeks of double-blind olanzapine or placebo	The results suggest that continued therapy with olanzapine may sustain and build upon improvements seen with acute olanzapine treatment of patients with BPD

Moen et al. (109)	A placebo-controlled study	17	All patients received dialectical behavior therapy for 4 weeks, then assigned into two groups; one received placebo and the other received divalproex ER for 12 weeks	There was a significant improvement in both groups from baseline. However, there was no advantage observed for divalproex ER and DBT over placebo and DBT

Schmahl et al. (110)	Two double-blind placebo-controlled randomized trials	25	Patients received both 3 weeks of naltrexone (50 or 200 mg/day) and 3 weeks of placebo in a randomized order	The dissociative symptoms were numerically not statistically significant lower under naloxone than placebo

Carrasco et al. (111)	A preliminary open-label study	49 patients with refractory BPD	The initial dose of 37.5 mg IM injection of LA risperidone repeated every 2 weeks, which could be raised to 50 mg for 6 months	IM risperidone may be effective and safe in patients with refractory BPD

Kröger et al. (112)	An open label study	1,423 patients with BPD	Dialectical behavior therapy	The response rate was 45%, 31% remained unchanged, and 11% deteriorated. Approximately 15% showed a symptom level equivalent to that of the general population

Jørgensen et al. (113)	A randomized controlled study	85 patients with BPD	2 years of intensive (twice weekly) combined (individual and group), mentalization-based psychotherapy or 2 years of less-intensive (biweekly) supportive group therapy	Significant changes in both treatment groups were identified for several outcome measures

Reneses et al. (114)	A randomized and controlled trial	44 patients	Psychic representation, focused psychotherapy, or treatment as usual	Results showed significantly better outcomes for the experimental group in all the main variables measured and in most of the secondary ones

Gratz et al. (115)	A randomized controlled trial and uncontrolled 9-month follow-up	61 patients	Adjunctive emotion regulation group therapy for 14 weeks	Results revealed significant improvements from pre- to posttreatment on all outcomes

Black et al. (116)	A randomized, double-blind, placebo-controlled trial	95 patients with BPD	150 mg/day of quetiapine (the low-dosage group), 300 mg/day of quetiapine (the moderate-dosage group), or placebo	Participants treated with 150 mg/day of quetiapine had a significant reduction in the severity of borderline personality disorder symptoms compared with those who received placebo. Adverse events were more likely in participants taking 300 mg/day of quetiapine

Harned et al. (117)	A pilot randomized controlled trial	26 with recent and recurrent intentional self-injury	DBT or DBT with the DBT Prolonged exposure	Patients who completed the DBT PE protocol were 2.4 times less likely to attempt suicide and 1.5 times less likely to self-injure than those in DBT

Linehan et al. (118)	A single blind randomized clinical trial and component analysis	99 women who had at least two suicide attempts and/or non-suicidal self-injury (NSSI) acts in the last 5 years, an NSSI act or suicide attempt in the 8 weeks before screening, and a suicide attempt in the past year	The study compared standard DBT, DBT-S, and DBT-I for 1 year and follow-up for another year	All treatment conditions resulted in similar improvements in the frequency and severity of suicide attempts, suicide ideation, use of crisis services due to suicidality, and reasons for living

Leichsenring et al. (119)	A randomized controlled efficacy-effectiveness study	179 patients	Manual-guided psychoanalytic-interactional therapy or non-manualized psychodynamic therapy by experts in personality disorders or placebo	Both PIT and E-PDT achieved significant improvements in all outcome measures and were superior to the control condition

## Introduction

The Diagnostic and Statistics Manual for Mental Disorders, fifth edition (2) classifies borderline line personality disorder (BPD) as a cluster B personality disorder and describes it as “a pervasive pattern of instability of interpersonal relationships, self-image, and affects, and marked impulsivity that begins by early adulthood and is present in a variety of contexts” (2). The Genesis of BPD is multifactorial, the biological inheritance, psychological, and social factors are the three major reasons for the development of BPD (3–7). Race, gender, and being socially disadvantageous influence the development of BPD (6–14). Functional impairment is the prime concern associated with the disease (15). BPD was once considered as an untreatable disease; however, the study by Gunderson and colleagues reported a remission rate of about 45% in 2 years and 85% in 10 years, indicating that correct diagnosis, proper, and timely management can allow the patient to live a normal life (15, 16).

Borderline personality disorder is a frequent psychiatric condition encountered in both the hospital and in psychiatric emergencies (17). Approximately 9–27% of agitated emergency patients are diagnosed with the borderline disorder (3, 18, 19). Predominantly, BPD patients visit an ED in the state of crisis, which includes immediate episodes of self-harm, suicidal attempt, aggressiveness, impulsivity, intense anxiety, short-term hallucinations, and delusions (17, 20). Such crises are usually short-lived, but severe in nature, and the intensity varies from person to person. Once the patient has reached the ED, the crisis state is either in the continuation or has subsided keeping the patient in a phase of strong emotional stress, which makes them non-cooperative. With such a heightened stress and difficult situation in the ED, identifying the disease, managing the patient, and defining the course of treatment becomes challenging not only for the attending psychiatrist but also for the accompanying staff. We review the difficulties faced by ED staff including physicians when diagnosing these patients, implementing a treatment regimen.

## Diagnostic Difficulty

Accurate diagnosis of the disease is necessary for deciding the future treatment regimen. A patient qualifies as BPD if he or she meets the five criteria out of the nine mentioned in DSM-5 (2). These criteria are: (1) frantic efforts to avoid abandonment; (2) a history of unstable and intense relationships with others; (3) identity disturbance; (4) impulsivity in at least two functional areas such as spending, sex, substance use, eating, or driving; (5) recurrent suicidal threats or behaviors as well as self-mutilation; (6) affective instability with marked reactivity of mood; (7) chronic feelings of emptiness; (8) inappropriate and intense anger or difficulty controlling anger; and (9) transient stress induced paranoid ideation or severe dissociative symptoms. This allows for significant variations in symptoms presentation from one BPD patient to another. Moreover, with overlapping clinical features with bipolar disease, pinpointing BPD becomes even more difficult (12). Although presenting symptoms of both the diseases are similar, their treatment course is completely different (21). The situation is further worsened when the patient in crisis displays a disruptive behavior and is non-cooperative. Thus, physicians are confronted with diagnostic dilemma and frustration. In such a situation, the crisis should be managed to make the patient more cooperative which, in turn, will aid an accurate identification of the disease and deciding the treatment course.

## Crisis Intervention in the EDs and Beyond

In a recent Cochrane review, Borschmann et al. defined crisis intervention as “an immediate response by one or more individuals to the acute distress experienced by another individual, which is designed to ensure safety and recovery and lasts not longer than 1 month” (22). Indeed, the priority of the ED physician who encounters a patient with BPD is to address any acute symptoms of distress and to calm the patient. However, the procedure for such crisis intervention is subjective.

In severely agitated patients, calming down them should be the prime focus. Managing agitation can be achieved by different methods such as administering non-specific sedating medication (benzodiazepines and/or antipsychotics), behavioral management, and psychological techniques such as de-escalation or physical restraint (23). Conventionally, benzodiazepines and antipsychotics are administered to control the agitated BPD patients. However, benzodiazepines use may lead to several side effects along with the strong sedation, hypotension, include respiratory depression, while typical antipsychotics cause dysphoria, acute dystonia, and akathisia. Recently, a few studies have reported that atypical antipsychotics such as Olanzapine, Ziprasidone, and Loxapine are more effective for treating acute crisis in BPD patients presenting to the ED (24–29). Intramuscular administration of single dose of olanzapine resulted in fast reduction of agitation in BPD patients and was tolerated well, with only 16% of patients requiring a second dose (25). Roncero and colleagues have shown the effectiveness of lopaxine in managing agitation in the emergency. In their study, inhalation of a single dose of lopaxine (9.1 mg) was enough to calm the acutely agitated patients (29). Prescribing mood stabilizers for managing agitation in BPD patients is highly discouraged by many studies (27, 30). In a report by Clivaz et al., administration of mood stabilizer topiramate augmented the incidences of panic attacks, thus worsening the situation (27). The authors recommended usage of atypical antipsychotics for managing the agitation in BPD patients (27).

In addition to agitation, the patient with BPD may present with a wide array of symptoms that indicate affective dysregulation. Such symptoms may include intense anger, mood liability, intense, depressive moods (31, 32). Administering high doses of antidepressant such as fluoxetine, a selective serotonin reuptake inhibitor is recommended. However, if it fails, then antidepressants targeting multiple neurotransmitters such as venlafaxine or monoamine oxidase inhibitors should be given. Additionally, BPD patients may also present with the elevated level of anxiety, administration of Clonazepam has shown to be effective in reducing anxiety (31, 32). However, Alprazolam, a drug belonging to the same class as clonazepam, has been reported to aggravate a hostile response.

In patients whose crisis state has subsided by arrival to the ED but who are experiencing severe emotional stress, such as suicidal attempters, psychotherapy is the preferred treatment option (32, 33). In this situation, psychotherapy should be relatively intense for short duration and discontinued before dependence on the therapist develops. Intensive DBT consisting of individual therapy sessions with an emphasis on skills training provided in groups, mindfulness skills, and team consultation for 3 weeks, have been shown to be effective for patients with BPD in crisis, especially with suicidal attempts or gestures (34, 35). Many studies nowadays suggest that after the crisis intervention, these patients should be continued on other form of psychotherapy such as; schematherapy (ST) and mentalization-based treatment (MBT). Because of the effectiveness and validation of MTB by numerous clinical studies, at present, it is widely suggested and included in treatment guidelines of BPD. Moreover, studies have found that intensive outpatient MBT effects are superior to conventional treatment (36, 37). Similarly, ST is a psychotherapy model integrating cognitive, experiential, and behavioral interventions together, which is remarkably effective in decreasing severe symptoms of BPD (38).

## Risk Management

Patients with BPD often present with any number of behaviors that are considered disruptive, such as self-harming injuries, violent behavior, impulsivity, or suicide. Such behavioral tendencies put the patient at significant risk to themselves and others if left unmanaged. Thus, the treatment of a patient with BPD should keep risk management in mind, especially as the patient nears the time of discharge. Efficient risk management in emergency settings is required for patients with BPD who exhibit self-injury, violent behavior, suicide, and impulsivity, all of which are considered displays of disruptive behavior (32). To achieve this understanding of the patient’s problem is of prime importance. However, patients and clinicians often have different opinions about the patient’s problem, in an emergency setting, due to the stress and chaos therein (39). Therefore, proper communication is necessary. Boggild et al. and Theinhaus et al. studied the factors responsible for disruptive behaviors in BPD patients. Both the groups found that patients with disruptive behaviors were significantly less likely to report the presence of primary support network, i.e., family members and had more work-related issues than a patient without disruptive behavior (39, 40). In this condition, developing a good rapport by proper communication and discussing their problems and finding alternative solutions should be the attitude of an attending clinicians, social workers, or nurses. Moreover, partial hospitalization has shown to be effective in managing crisis in BPD (41, 42). Nonetheless, the presence of general medical conditions as infectious diseases, psychosocial, and environmental problems such as housing problems, or chronic suicidal ideations are also associated with disruptive behaviors during hospitalization (39). Therefore, addressing the underlying causes of the patient’s disturbance should be approached as plainly such as treating a patient’s infectious disease: understand and address the reason for the suicide attempt and addressing them or finding a solution for temporary stay after discharging from the hospital, which provide comfort to the patients and helping to reduce their disruptive behavior.

## Approach Toward BPD Patients

Numerous studies have opined that clinicians and medical staff project a negative attitude for patients with BPD, more so for patients with self-damage or suicidal attitude (43–45). The main reasons for negative attitude include the stigma toward BPD, patients are considered as manipulative, lack of optimism for recovery, work pressure, poor communication skills, and time restraints (43, 46). Among the clinicians in psychiatric department, nurses exhibited negative approach as well as least compassion and hope for the recovery of these patients followed by psychiatrist and psychologist (44, 45, 47). Social workers showed the highest concern, compassion, and treatment optimism for BPD patients. Moreover, general hospital staff display a more adverse attitude toward BPD patients than the employees of psychiatric department (45).

The negative attitude toward BPD patient in crisis makes them more volatile, non-complaint, which makes the diagnosis and treatment difficult leading to problematic outcomes like unnecessary hospitalizations, improper safety assessments, unneeded use of medications, extreme use of physical restraints, and, ultimately, increased liability (44, 45, 47). Imparting proper education through training and workshops separately to different categories of employers in the ED and the general hospital has shown to be effective in building a positive attitude, compassion, and patience toward BPD patients (46, 48, 49). In the crisis, a BPD patient not only comes in contact with emergency medical staff but also with ambulatory staffs as they require ambulance service to arrive emergency (50). Therefore, training of ambulance staff is also required. Treolar et al. (51) assessed the effectiveness of cognitive behavioral treatment and psychoanalytics in changing the attitude toward BPD with self-harm attitude (46). Both treatments showed remarkable improvements in the attitude of clinicians and medical staffs and the effect of psychonalytics was long lasting.

Considering the above studies, applying psychonalytics to different categories of hospital employers starting from ambulance staff to psychiatric and general emergency staff and clinicians for educating them about BPD should be carried out for better management and recovery in these patients.

## Treatment

Subsequent to crisis control, proper diagnosis and a treatment regimen should be addressed. The aim of treatment should be to decrease the severity in symptoms such as self-damage, suicidal attempts/gesture, impulsivity, aggressiveness, substance abuse, etc., which in turn will reduce the number of visits to the ED. The treatment for BPD usually lasts for months or years. Psychotherapy along with pharmacotherapy is the usual mode of treatment for BPD (32, 52, 53). Cognitive behavioral therapy (CBT) and dialectical behavior therapy (DBT) are two most extensively studied forms of psychotherapy in BPD patients.

When considering treatment options, physicians should be aware that CBT is an effective and affordable addition to existing care. Davidson et al. assessed the effectiveness of addition of CBT to treatment-as-usual for 1–2 years and reported that adding CBT has only small effect as the number of hospitalization and emergency visits were comparable between both the groups (54). The same group also assessed the long-term effect of the CBT (2–6 years) over treatment-as-usual (55). They observed a decreased in a number of suicidal attempts only. Nevertheless, measures of depression, anxiety, general psychopathology, social functioning, quality of life, dysfunctional attitudes, emergency visits, and mean length of hospitalization were comparable between both the groups. The same group had also utilized a variation of CBT, manual assisted CBT, for treating BPD and concluded that it was unable to reduce the number of attempts for self-damage BPD and was not cost-effective (56).

Linehan et al. conducted an RCT wherein they subjected the BPD with suicidal attempts to DBT for 2 years and observed that it was efficient in reducing the suicide attempt, length of hospitalization for suicide ideation, lowered the medical risk, and decreased psychiatric ED visits (57). Similarly, in adolescents, application of DBT for 52 weeks showed a robust long-term decrease in incidences of self-damage and a fast recovery in suicidal ideation, depression, and borderline symptoms (58, 59). Application of Combined Individual and Group DBT, a variation of DBT, for 12 and 18 months, was found to be equivalent to DBT in reducing the number of suicide attempts, suicidal behavior, and number of emergency visits (60).

Mentalization-based therapy has become a promising psychodynamic approach and added into guidelines for the treatment BPD patients. Mentalizing is related to the capability of interpreting self and others in the form of emotions, feelings, desires, and values. Studies suggest that mentalization impairments are associated with BPD (41). MBT effectively reduces depressive symptoms, suicidal attempts, and self-harm, which also include increasing social functioning in BPD patients (61). As we described earlier in introduction, ST offers an effective help, shows a significant improvement of core symptoms of personality disorder. Reiss et al. summarized the results of three pilot studies investigating the effect of intensive inpatient ST program delivered in individual or group format. Results showed that inpatient ST can significantly reduce symptoms of severe BPD (38). Similarly, other studies have found a large treatment effect in reduction in severity of BPD, impulsivity, affective instability, and general psychopathology (62, 63).

Lana et al. adopted an integrated approach to treat severe BPD patients, which uses multiple psychotherapies in one treatment session (64). It included skill training group based on DBT; relationship therapy supported by MBT; stress management group; and psychoeducational group; individual therapy once a week, support by psychodynamic psychotherapy or DBT depending on the clinician’s approach; medication review; nursing consultation; and telephone consultation for 6 months. Patients with integrated treatment had a lower number of visits to ED and decreased the length of hospitalization during the treatment duration as well as beyond it indicating in efficiency in treating BPD.

A recent systematic review analyzed the competency of psychotherapies in scaling down the suicidal attempts and non-suicidal self-injury (NSSI) in borderline patients (65). It concluded that psychotherapy seems to be an effective treatment for suicidal attempts only. For NSSI cases, MBT was a better means of management. This raises a question of the applicability of psychotherapy in BPD patients with other severity symptoms. Hence, a better means of treatment with the broad application is needed. In recent years, functional neuroimaging research describes BPD patients with the dysfunctional frontolimbic network (66). Further, severe BPD patients have impairment in decision-making functionality (67). In view of the above findings, Cailhol et al. evaluated the advantages of intermittent application of high-frequency transcranial magnetic stimulation (TMS) application on the right cerebral cortex in treating BPD (68).

Transcranial magnetic stimulation remarkably reduced the anger and affective instability in BPD patients after 3 months indicating as a promising technique for managing and treating BPD. As this technique is safe with no side effects and is applied intermittently, its application in acute crisis interventions should also be examined.

## Conclusion

Borderline personality disorder patients in crisis are frequent visitors of EDs. Due to lack of knowledge on BPD and social stigma, emergency clinicians and staff develop a negative approach for these patients, which in turn have a negative impact on their treatment outcome. Clinicians and medical staffs should be properly educated about this disorder, which will aid in getting into a comfortable zone with these patients, develop compassion for them and ways for managing the crisis. There is lack of RCTs investigating the efficacy of crisis interventions for people with BPD. Although psychotherapies show a positive effect on reducing BPD-related symptoms, the effect is small. Therefore, we recommend conducting prospective high-quality clinical trials with balanced control groups. These trials should measure a wider range of primary and secondary outcomes of the treatment investigated. All this together will help the patient to get back to the pre-crisis phase and make them more receptive to the actual treatment process.

## Author Contributions

SA has provided this idea to write about this important topic, supervised this manuscript, edited its grammar, and added references, written conclusion. US has helped to write manuscript, especially the introduction section. IQ has helped tremendously in this paper, she has worked hard to write two important section of papers including literature search and assisting in finding hand-pick papers from the library. FJ has helped to write a section of crisis intervention, her clinical experience was utilized to finalized that section. SS has helped us to add all studies provided in the table. She also corrected grammar and syntax of the language. YO helped to fix reference, discussion section.

## Conflict of Interest Statement

The authors declare that the research was conducted in the absence of any commercial or financial relationships that could be construed as a potential conflict of interest.
